# Production of a human milk oligosaccharide 2′-fucosyllactose by metabolically engineered *Saccharomyces cerevisiae*

**DOI:** 10.1186/s12934-018-0947-2

**Published:** 2018-06-27

**Authors:** Sora Yu, Jing-Jing Liu, Eun Ju Yun, Suryang Kwak, Kyoung Heon Kim, Yong-Su Jin

**Affiliations:** 1 Department of Biotechnology, Graduate School, Korea University, Seoul, 02841 South Korea; 2 Carl R. Woese Institute for Genomic Biology, University of Illinois at Urbana-Champaign, Urbana, IL 61801 USA; 3 Department of Food Science and Human Nutrition, University of Illinois at Urbana-Champaign, Urbana, IL 61801 USA

**Keywords:** 2′-Fucosyllactose, Human milk oligosaccharide, *Salvage* pathway, α-1,2-Fucosyltransferase, Lactose permease, *Saccharomyces cerevisiae*

## Abstract

**Background:**

2′-Fucosyllactose (2-FL), one of the most abundant oligosaccharides in human milk, has potential applications in foods due to its health benefits such as the selective promotion of bifidobacterial growth and the inhibition of pathogenic microbial binding to the human gut. Owing to the limited amounts of 2-FL in human milk, alternative microbial production of 2-FL is considered promising. To date, microbial production of 2-FL has been studied mostly in *Escherichia coli*. In this study, 2-FL was produced alternatively by using a yeast *Saccharomyces cerevisiae*, which may have advantages over *E. coli*.

**Results:**

Fucose and lactose were used as the substrates for the *salvage* pathway which was constructed with *fkp* coding for a bifunctional enzyme exhibiting l-fucokinase and guanosine 5′-diphosphate-l-fucose phosphorylase activities, *fucT2* coding for α-1,2-fucosyltransferase, and *LAC12* coding for lactose permease. Production of 2-FL by the resulting engineered yeast was verified by mass spectrometry. 2-FL titers of 92 and 503 mg/L were achieved from 48-h batch fermentation and 120-h fed-batch fermentation fed with ethanol as a carbon source, respectively.

**Conclusions:**

This is the first report on 2-FL production by using yeast *S. cerevisiae*. These results suggest that *S. cerevisiae* can be considered as a host engineered for producing 2-FL via the *salvage* pathway.

**Electronic supplementary material:**

The online version of this article (10.1186/s12934-018-0947-2) contains supplementary material, which is available to authorized users.

## Background

Human milk oligosaccharides (HMOs) are important components of human milk that promote infant health [1]. Fucosylated oligosaccharides, one of the most common HMOs, have been reported to offer health benefits, such as selectively enhancing bifidobacterial growth and effectively preventing binding of pathogens and toxins to the human gut [2, 3]. In particular, 2′-fucosyllactose (2-FL), the most abundant fucosylated oligosaccharide in human milk, attracted much interest as a functional food ingredient [4] because of its nutraceutical and pharmaceutical properties [5].

Owing to the scarce contents of 2-FL in human milk, it is prohibitively expensive to obtain 2-FL directly from human milk [6, 7]. Alternatively, production of 2-FL using metabolically engineered microorganisms has been attempted [8, 9]. Production of 2-FL requires α-1,2-fucosyltransferase which transfers the fucosyl residue from guanosine 5′-diphosphate-l-fucose (GDP-l-fucose) into lactose. GDP-l-fucose can be generated through two distinct metabolic pathways: the de novo or *salvage* pathway. In the de novo pathway, GDP-l-fucose is synthesized from mannose-6-phosphate by GDP-mannose 4,6-dehydratase and GDP-l-fucose synthase. The alternative *salvage* pathway requires l-fucose as the substrate for producing GDP-l-fucose. This pathway is catalyzed by a bifunctional enzyme, l-fucokinase/GDP-l-fucose phosphorylase (FKP). The *salvage* pathway was assumed to exist only in eukaryotes until a bacterial FKP was discovered from *Bacteroides fragilis* 9343 [10].

Previous studies of metabolic engineering to produce 2-FL have mostly been conducted using *Escherichia coli* as a host. To supply ample amounts of GDP-l-fucose, either the existing de novo pathway was augmented, or the *salvage* pathway was introduced into *E. coli* [9, 11–15]. Through a fed-batch fermentation using engineered *E. coli* harboring the *salvage* pathway genes, 2-FL was produced at a concentration of 23.1 g/L with yields of 0.367 mol 2-FL/mol lactose and 0.355 mol 2-FL/mole fucose [14].

To avoid possible endotoxin contamination in the produced 2-FL, and bacteriophage infection in the fermentation process to produce 2-FL using engineered *E. coli*, this study aimed to produce 2-FL by engineered *Saccharomyces cerevisiae* which is generally recognized as safe (GRAS) and has been widely used in food and pharmaceutical industries. Intracellular production of GDP-l-fucose in engineered *S. cerevisiae* has been achieved through introducing the de novo and *salvage* pathways [16–18]. An engineered *S. cerevisiae* strain was able to produce 0.13 mg of GDP-l-fucose from 25 mL culture containing 2.46 g/L of l-fucose via the *salvage* pathway [16]. However, 2-FL production in engineered yeast via the de novo or *salvage* pathway has not been reported yet.

**Fig. 1:**
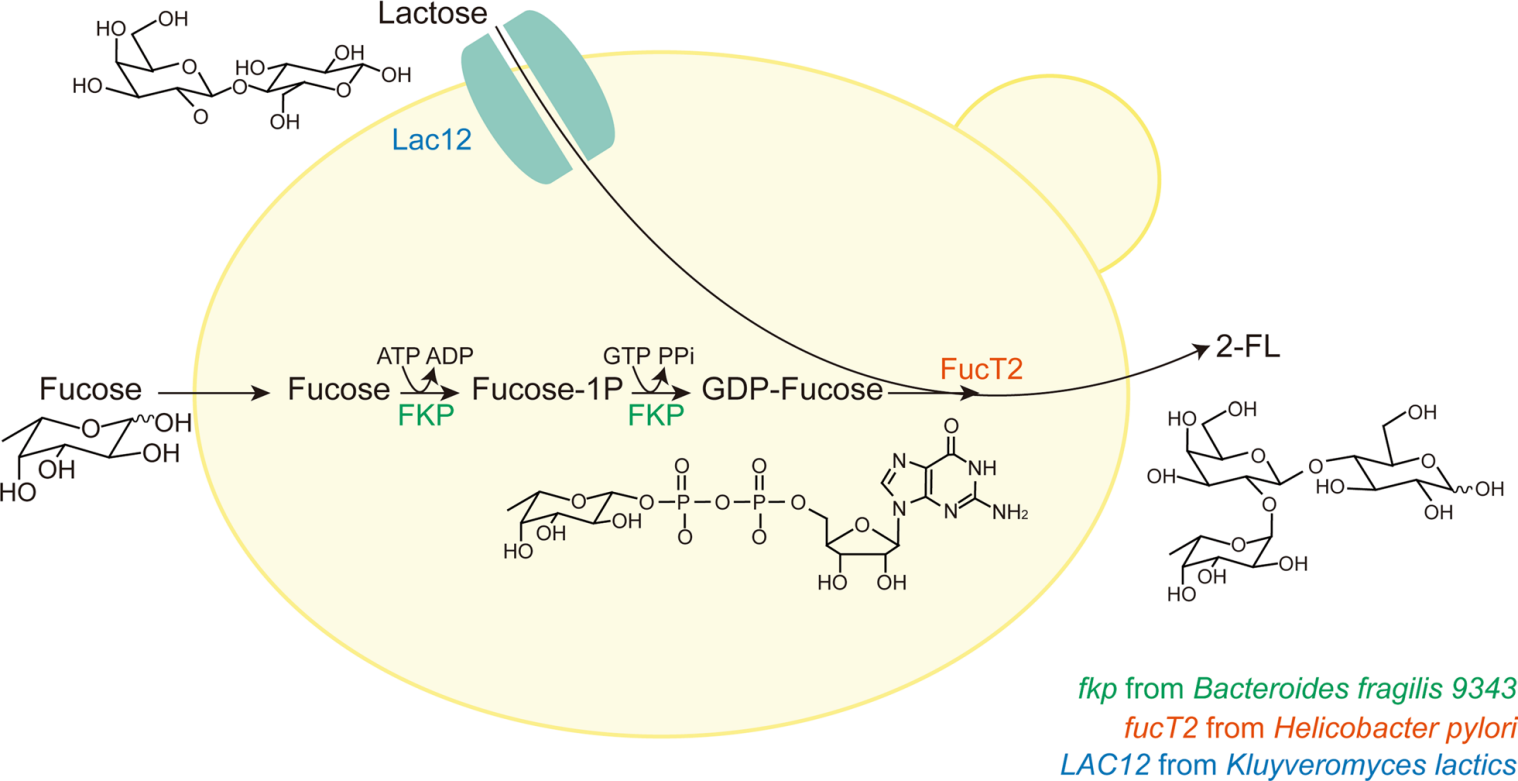
Schematic representation of 2′-fucosyllactose production by engineered yeast *Saccharomyces cerevisiae*. *fkp*: the gene coding for l-fucokinase/GDP-l-fucose phosphorylase (FKP); *fucT2*: the gene coding for α-1,2-fucosyltransferase; and *LAC12*: the gene coding for lactose permease

To produce 2-FL in engineered *S. cerevisiae* via the *salvage* pathway using l-fucose and lactose as substrates, at least three genetic modifications must be achieved (Fig. 1). First, FKP needs to be introduced to produce intracellular GDP-l-fucose as a substrate of fucosyltransferase. Intracellular GDP-l-fucose level has been considered as a bottleneck in 2-FL production [9, 11]. It was shown that the productivity of 2-FL increased by over 80% by introducing the *salvage* pathway in addition to the de novo pathway in engineered *E. coli* [11]. Second, lactose, a fucose acceptor in 2-FL production, needs to be transported into the cytosol of *S. cerevisiae* cells. Lactose permease, such as Lac12 from *Kluyveromyces lactics* [19] or CDT-1 from *Neurospora crassa* [20, 21] needs to be introduced into *S. cerevisiae* for transporting lactose into the cytosol because wild-type *S. cerevisiae* is incapable of transporting lactose into the cytosol. Finally, α-1,2-fucosyltransferase, which catalyzes fucosylation of lactose into 2-FL using GDP-l-fucose needs to be introduced into *S. cerevisiae*. Several α-1,2-fucosyltransferases have been verified to facilitate the synthesis of 2-FL, which included FucT2 from *Helicobacter pylori* [22, 23], WcfB from *B. fragilis* 9343 [13, 15], and WbgL from *E. coli* O126 [13, 24]. Among those, FucT2 has been most frequently used for the microbial production of 2-FL [9, 11–14].

In this study, *S. cerevisiae* was engineered to produce 2-FL via the *salvage* pathway using l-fucose and lactose as the substrates for producing 2-FL. First, overproduction of GDP-l-fucose was examined by expressing three different FKPs from *Bacteroides* species, including *B. fragilis* 9343. Second, an α-1,2-fucosyltransferase (FucT2) from *H. pylori* and a lactose permease (Lac12) from *K. lactics* were introduced into a GDP-l-fucose accumulating strain to produce 2-FL. Finally, 2-FL produced in the engineered yeast was verified by mass spectrometry, and fermentation conditions were modified to increase titers of 2FL. To our knowledge, this is the first study of 2-FL production using yeast as a host.

## Methods

### Strains, plasmids, and yeast transformation

Genes coding for FKP were from three *Bacteroides* species, namely, *B. fragilis* 9343, *B. thetaiotaomicron*, and *B. ovatus* (Table 1). The *fucT2* gene from *H. pylori* was codon-optimized for the expression in *S. cerevisiae* and synthesized by Integrated DNA Technologies (Coraville, IA, USA). *LAC12* coding for lactose permease was amplified from the genomic DNA of *K. lactics* (Table 1). These genes were inserted into the plasmids pRS425GPD, pRS426GPD, or pRS423GPD [25]. *E. coli* TOP10 (Invitrogen, Carlsbad, CA, USA) was used for construction and manipulation of plasmids (Table 1). *S. cerevisiae* D452-2 (*MATα, leu2, his3, ura3, and can1*) [26] was used as the host for producing 2-FL (Table 2). Plasmids were transformed into *S. cerevisiae* by the lithium acetate/single-stranded carrier DNA/polyethylene glycol method as described previously [27].

**Table 1:** Primers and plasmids used or constructed in this study

Name	Description	Reference
Primer	Sequence (restriction sites are underlined)	
F_BF_*fkp* (*Bam*HI)	ATAGGATCCATGCAAAAACTACTATCTTTGCCTCCTAATC	This study
R_BF_*fkp* (*Sal*I)	ATAGTCGACTTATGATCGTGATACTTGGAATCCCTTATCCG	This study
F_BT_*fkp* (*Bam*HI)	ATAGGATCCATGCCGGAGCCGATCTGCTGTTTCCTTC	This study
R_BT_*fkp* (*Sal*I)	ATAGTCGACTTAGCTTCTCGATACCTGTAATCC	This study
F_BO_*fkp* (*Bam*HI)	ATAGGATCCATGCAAAAGTTATTATCCTTACCAC	This study
R_BO_*fkp* (*Sal*I)	ATAGTCGACTTAGCTTCTTGAAACCTGAAGTCCCTTGTCAG	This study
F_*LAC12* (*Spe*I)	TCTAGAGCGGCCGCACTAGTGCCACCATGGCAGATCATTCGAGCAG	This study
R_*LAC12* (*Sal*I)	TCTAGAGCGGCCGCGTCGACTTAAACAGATTCTGCCTCTG	This study
F_*fucT2* (*Spe*I)	GG ACTAGTATGGCCTTTAAGGTCGT	This study
R_*fucT2* (*Xho*I)	CCGCTCGAGTTAGGCATTATACTTTTGAGACTTAACT	This study
Plasmid		
pRS423GPD	*HIS3*, *GPD* promoter, *CYC1* terminator, 2 µ origin, and Amp^r^	[25]
pRS425GPD	*LEU2, GPD* promoter, *CYC1* terminator, 2 µ origin, and Amp^r^	[25]
pRS426GPD	*URA3, GPD* promoter, *CYC1* terminator, 2 µ origin, and Amp^r^	[25]
pRS425GPD_BF_*fkp*	pRS425GPD harboring *fkp* from *B. fragilis 9343*	This study
pRS425GPD_BT_*fkp*	pRS425GPD harboring *fkp* from *B. thetaiotaomircon*	This study
pRS425GPD_BO_*fkp*	pRS425GPD harboring *fkp* from *B. ovatus*	This study
pRS423GPD_*LAC12*	pRS425GPD harboring *LAC12* from *K. lactics*	This study
pRS426GPD_*fucT2*	pRS425GPD harboring *fucT2* from *H. pylori*	This study

**Table 2:** Strains used or constructed in this study

Name	Description	Reference
D452-2	*S. cerevisiae, MATα, leu2, his3, ura3, and can1*	[26]
D452-2_FKP_Control	D452-2 harboring pRS425GPD	This study
D452-2_BF_FKP	D452-2 harboring pRS425GPD_BF_*fkp*	This study
D452-2_BT_FKP	D452-2 harboring pRS425GPD_BT_*fkp*	This study
D452-2_BO_FKP	D452-2 harboring pRS425GPD_BO_*fkp*	This study
D452-2_LFF	D452-2 harboring pRS423GPD_*LAC12*, pRS425GPD_BF_*fkp*, and pRS426GPD_*fucT2*	This study
D452-2_LFF_Control	D452-2 harboring pRS423GPD, pRS425GPD, and pRS426GPD	This study

### Culture media and conditions

*E. coli* strains were grown in Luria–Bertani medium containing 100 µg/mL ampicillin at 37 °C and 200 rpm for plasmid amplification. After yeast transformation, yeast synthetic complete (YSC) medium was used, which contained 6.7 g/L yeast nitrogen base with 20 g/L glucose, 20 g/L agar, and 0.69 g/L CSM-Leu (MP Biomedicals, Solon, OH, USA) or 0.65 g/L CSM-His-Leu-Ura (MP Biomedicals), which supplied appropriate nucleotides and amino acids to select transformants using auxotrophic markers.

To verify the accumulation of GDP-l-fucose by expressing FKP in engineered yeast, three engineered *S. cerevisiae* strains, D452-2_BF_FKP harboring *fkp* from *B. fragilis* 9343, D452-2_BT_FKP harboring *fkp* from *B. thetaiotaomicron*, D452-2_BO_FKP harboring *fkp* from *B. ovatus*, and D452-2_FKP_Control harboring an empty vector, were grown in the YSC medium containing 6.7 g/L yeast nitrogen base with 20 g/L glucose, 0.69 g/L CSM-Leu, 5 g/L fucose, and 2 mM MgCl_2_ in 50 mM potassium hydrogen phthalate (KHP) buffer (pH 5.5) at 30 °C and 250 rpm for 36 h. Initial cell densities were adjusted to optical density at 600 nm (OD_600_) = 0.1.

To examine 2-FL production in the engineered yeast (D452-2_LFF) expressing three heterologous genes (*fkp, fucT2, and LAC12*), batch fermentation was performed in a 50-mL flask containing 10 mL of a modified Verduyn medium [28] with 20 g/L glucose as a carbon source for growth, and 2 g/L fucose and 2 g/L lactose as substrates for 2-FL production in 50 mM KHP buffer (pH 5.5) at 30 °C and 250 rpm. For this fermentation, initial cell densities were adjusted to OD_600_ = 1. As a control, batch fermentation of strain D452-2_LFF_Control harboring three different empty vectors was also performed under the same conditions.

### Fed-batch fermentation

To test the feasibility of producing 2-FL at high titers, fed-batch fermentation was performed. To prepare a seed culture, the engineered yeast, D452-2_LFF was cultivated overnight in a 50-mL flask containing 10 mL of the modified Verduyn medium with 20 g/L glucose in 50 mM KHP buffer (pH 5.5) at 30 °C and 250 rpm. Cells from the seed culture were harvested by centrifugation at 1789×*g* for 5 min at 4 °C. For the fed-batch fermentation, the cells obtained by the centrifugation were inoculated into a 125-mL baffled flask containing 25 mL of the modified Verduyn medium containing 20 g/L glucose, 2 g/L fucose, and 2 g/L lactose in 50 mM KHP buffer (pH 5.5). The initial cell density was set at OD_600_ = 1, and the fermentation was performed at 30 °C and 250 rpm. The fed-batch fermentation process was divided into the glucose batch phase and the ethanol feeding phase. In the glucose batch phase, 20 g/L glucose was added as the initial carbon source. After the initially added glucose and the produced ethanol from glucose during cultivation were depleted, 20 g/L ethanol was added into the flask as a carbon source. When the added ethanol was depleted, additional 20 g/L ethanol was fed into the flask until 120 h.

### Cell growth monitoring and quantification of 2-FL by high performance liquid chromatography

Cell growth was monitored by OD_600_ measurement using a UV–visible spectrophotometer (Biomate5; Thermo Fisher Scientific, Waltham, MA, USA).

To verify the production of GDP-l-fucose and to measure intracellular GDP-l-fucose concentrations in engineered yeast, 5 mL of cell culture was harvested by centrifugation at 1789×*g* for 5 min at 4 °C, washed with distilled water, and resuspended in 500 µL of distilled water. The cells were disrupted using glass beads for 1 h. After centrifugation at 9447×*g* for 10 min at 4 °C, 10 µL supernatant was injected into an high performance liquid chromatography (HPLC) system (Shimadzu, Kyoto, Japan) equipped with a CAPCELL PAK C18 MG column (250 × 4.6 mm, Shiseido, Tokyo, Japan) at 30 °C. The flow rate of a mobile phase composed of 20 mM triethylamine at pH 6 and 2% (v/v) acetonitrile was set at 0.6 mL/min. GDP-l-fucose was detected at 254 nm by HPLC, and the concentration of GDP-l-fucose was calculated from its peak area using the GDP-l-fucose standard (Carbosynth, Compton, UK). The concentrations of glucose, fucose, 2-FL, and ethanol were measured by an HPLC system (Agilent Technologies 1200; Agilent Technologies, Wilmington, DE, USA) equipped with a refractive index (RI) detector using a Rezex ROA-Organic Acid H^+^ (8%) column (Phenomenex, Torrance, CA, USA). The column and RI detector temperatures were set at 50 °C, and the column was eluted with 0.005 N of H_2_SO_4_ at a flow rate of 0.6 mL/min. The concentration of lactose was measured by the HPLC system (Agilent Technologies 1200) equipped with a RI detector using a Rezex RCM-Monosaccharide Ca^+2^ (8%) column (Phenomenex). The column and RI detector temperatures were set at 80 °C, and the column was eluted with water at a flow rate of 0.6 mL/min.

### Identification of 2-FL by gas chromatography/mass spectrometry and liquid chromatography/mass spectrometry

To identify 2-FL produced in the engineered yeast, culture broths were analyzed using gas chromatography/mass spectrometry (GC/MS) and liquid chromatography/mass spectrometry (LC/MS). For GC/MS analysis, culture broths obtained from batch fermentations of strains D452-2_LFF and D452-2_Control were centrifuged at 9447×*g* for 10 min, and 20 µL of supernatants were dried in a centrifugal vacuum evaporator. For chemical derivatization before GC/MS analysis, 10 µL of 40 mg/mL methoxyamine hydrochloride in pyridine (Sigma-Aldrich, St. Louis, MO, USA) was added to the dried samples, and incubated at 30 °C. After 90 min, 45 µL of *N*-methyl-*N*-(trimethylsilyl)-trifluoroacetamide (Sigma-Aldrich) was added and incubated for 30 min at 37 °C. The 2-FL standard (Carbosynth) was derivatized using the same method described above. The chemically derivatized samples were analyzed using an Agilent 7890A GC/5975C MSD system (Agilent Technologies) equipped with an HP-5 ms column (30 m in length, 0.25 mm in diameter, and 0.25 μm in film thickness; Agilent Technologies) and a 10-m guard column. The derivatized sample (1 µL) was injected into the GC column in splitless mode. The oven temperature was programmed to be initially at 80 °C for 1 min and then be ramped to 300 °C at 10 °C/min for 1 min. Electron ionization was performed at 70 eV, and the temperatures of the ion source and transfer line were 250 and 280 °C, respectively. The mass range used was 85–700 *m/z*.

To analyze culture broths of fed-batch fermentation, LC/MS ion-trap and time-of-flight system (Shimadzu) equipped with a Thermo Hypercarb porous graphitic carbon LC column (100 mm in length, 2.1 mm in diameter, and a 3 μm in particle size; Thermo Fisher Scientific) was used. The mobile phase was composed of solutions A (25 µM lithium chloride) and B (acetonitrile). The mass spectrometer was operated in positive ion mode. The injection volume of each sample was 20 µL. The gradient elution was from 0% (v/v) to 80% in 41 min, and the flow rate of the mobile phase was set at 0.2 mL/min. The temperatures of the LC column and the autosampler were set at 70 and 10 °C, respectively. Source-dependent parameters were set at as follows: nebulizing gas flow rate, 1.5 L/min; interface voltage, 4.5 kV; detector voltage, 1.65 kV; curved desolvation line temperature, 200 °C; and heat block temperature, 200 °C. The mass range used was 100–700 *m/z*.

### Quantification of extracellular and intracellular 2-FL from engineered yeast

Extracellular and intracellular 2-FL concentrations were quantified during fed-batch fermentation of the engineered *S. cerevisiae*. To measure extracellular 2-FL concentrations, cells were removed by centrifugation of fermentation broths at 1789×*g* for 5 min at 4 °C, and the supernatants were analyzed by HPLC to determine the concentrations of 2-FL. To determine intracellular 2-FL concentrations, intracellular metabolites containing 2-FL were enabled to be release outside cells by boiling fermentation broths for 10 min at 100 °C [29]. The boiled fermentation broths were centrifuged at 9447×*g* for 10 min at 4 °C, and the supernatants were analyzed by HPLC to measure 2-FL concentrations. The measured 2-FL concentrations of the supernatants were considered the total concentrations of 2-FL in the fed-batch flasks of the engineered yeast. To determine the intracellular 2-FL concentrations, the total 2-FL concentrations was subtracted by the extracellular 2-FL concentrations.

## Results

### GDP-l-fucose accumulation in engineered yeast

**Fig. 2:**
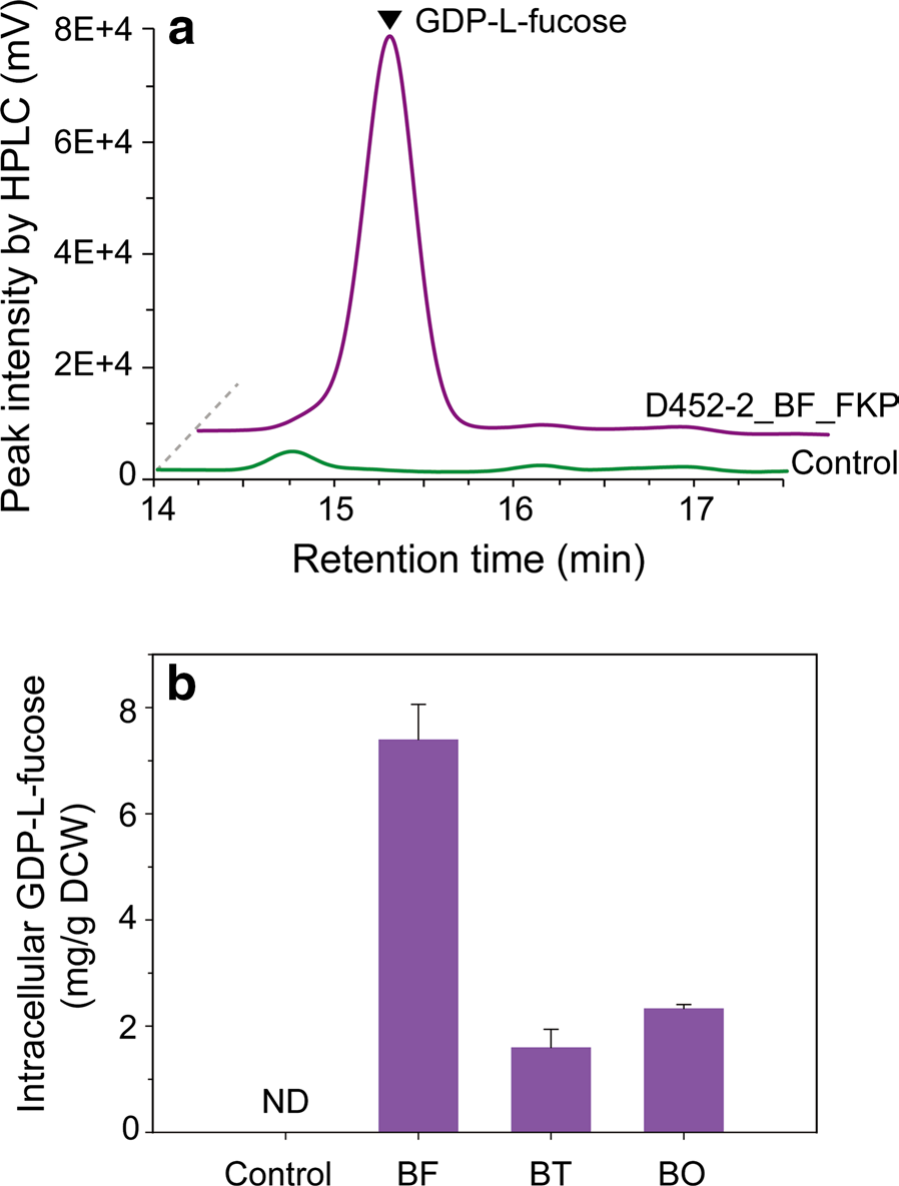
Production of 5′-diphosphate-l-fucose (GDP-l-fucose) in engineered *Saccharomyces cerevisiae*. Engineered strains cultured in yeast synthetic complete (YSC) medium composed of 6.7 g/L yeast nitrogen base with 20 g/L glucose, 0.69 g/L CSM-Leu, 5 g/L l-fucose, and 2 mM MgCl_2_ in 50 mM potassium hydrogen phthalate buffer (pH 5.5) at 30 °C and 250 rpm for 36 h. **a** The overlaid HPLC chromatograms of D452-2_BF_FKP and control strains. **b** Comparison of intracellular GDP-l-fucose concentrations depending on the origin of the gene, *fkp*, coding for l-fucokinase/GDP-l-fucose phosphorylase (FKP). Control (D452-2_FKP_Control), *S. cerevisiae* D452-2 harboring pRS425GPD; BF (D452-2_BF_FKP), *S. cerevisiae* D452-2 harboring pRS425GPD_BF_*fkp*; BT (D452-2_BT_FKP), *S. cerevisiae* D452-2 harboring pRS425GPD_BT_*fkp*; BO (D452-2_BO_FKP), *S. cerevisiae* D452-2 harboring pRS425GPD_BO_*fkp*; ND, not detectable; DCW, dry cell weight. All data points are the means of experimental data from duplicate fermentations

To produce 2-FL in engineered yeast, ample supply of GDP-l-fucose is necessary for fucosylation of lactose by α-1,2-fucosyltransferase. To enable accumulation of GDP-l-fucose in the cytosol, genes coding for FKP originating from 3 different *Bacteroides* species were individually introduced into *S. cerevisiae* D452-2. When the engineered yeast D452-2_BF_FKP overexpressing FKP from *B. fragilis* 9343 was cultured in the presence of fucose, a significant peak at an approximate retention time of 15.1 min was detected in the sample (Fig. 2a). In contrast, no peak was detected in the sample from the control strain, D452-2_FKP_Control harboring the empty vector.

Regardless of the origins of *fkp* genes in this study, the production of GDP-l-fucose by the engineered yeasts occurred. However, the amount of GDP-l-fucose produced in strain D452-2_BF_FKP overexpressing FKP from *B. fragilis* 9343 was higher than that in strains D452-2_BT_FKP and D452-2_BO_FKP overexpressing each FKP from *B. thetaiotaomicron* and *B. ovatus* by 4.6 and 3.2 times, respectively (Fig. 2b). These results indicate that all three genes coding for FKP originating from *B. fragilis* 9343, *B. thetaiotaomicron*, and *B. ovatus* were functionally expressed, which enabled the yeast to produce GDP-l-fucose intracellularly. However, different concentrations of intracellular GDP-l-fucose were measured depending on the origin of genes coding for FKP. Thus, *fkp* from *B. fragilis* 9343, which showed the highest concentration of intracellular GDP-l-fucose, was selected and introduced into *S. cerevisiae* D452-2 for 2-FL production.

### Production and identification of 2-FL in the engineered yeast

**Fig. 3:**
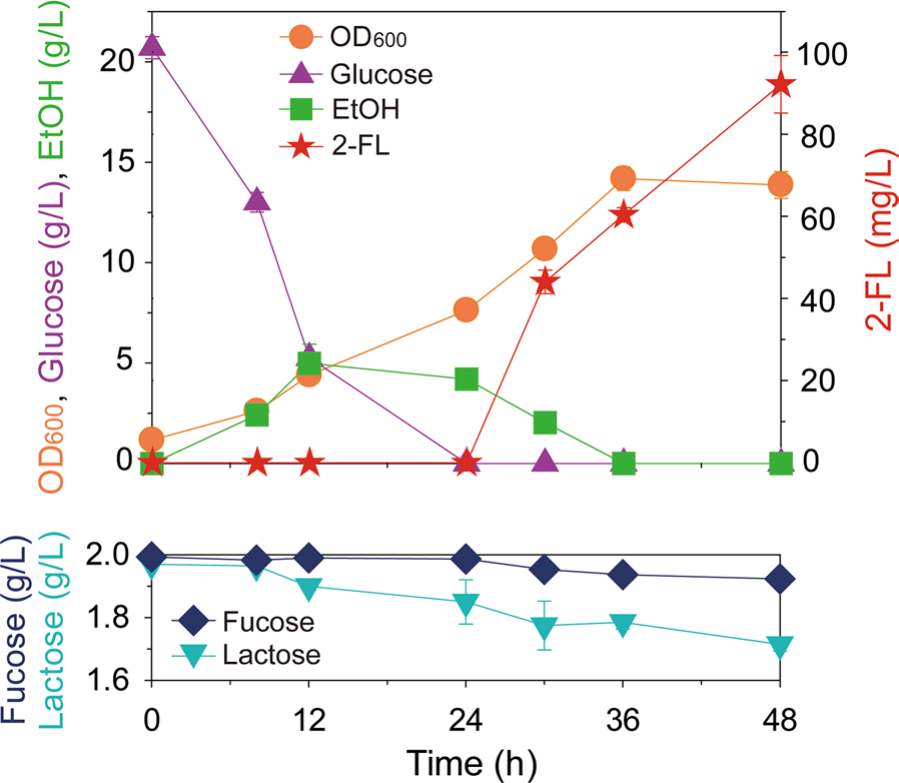
Batch fermentation profiles of engineered *Saccharomyces cerevisiae* D452-2_LFF in a modified Verduyn medium with 20 g/L glucose, 2 g/L fucose, and 2 g/L lactose in 50 mM potassium hydrogen phthalate buffer (pH 5.5) at 30 °C and 250 rpm for 48 h. During fermentation, the cell density (OD_600_) and concentrations of glucose, ethanol (EtOH), and 2′-fucosyllactose (2-FL) were monitored by HPLC. All data points are the means of experimental data from duplicate fermentations. Strain D452-2_LFF indicates *S. cerevisiae* D452-2 harboring *fkp* coding for l-fucokinase/guanosine 5′-diphosphate-l-fucose phosphorylase, *fucT2* coding for α-1,2-fucosyltransferase, and *LAC12* coding for lactose permease

To produce 2-FL via the *salvage* pathway in engineered yeast, three heterologous genes (*fkp*, *fucT2*, and *LAC12*) coding for *B. fragilis* 9343 FKP, *H. pylori* α-1,2-fucosyltransferase, and *K. lactics* lactose permease, respectively, were overexpressed in *S. cerevisiae* D452-2. To confirm 2-FL production in the resulting yeast strain (D452-2_LFF), flask cultures were performed. Initially added glucose and produced ethanol during glucose fermentation were completely consumed after 36 h, and 92 mg/L of 2-FL was produced after 48 h (Fig. 3). Until 48 h, 70 mg/L of l-fucose and 285 mg/L of lactose were consumed. Thus, the yields of 2-FL were 0.44 mol/mol from l-fucose and 0.25 mol/mol from lactose.

**Fig. 4:**
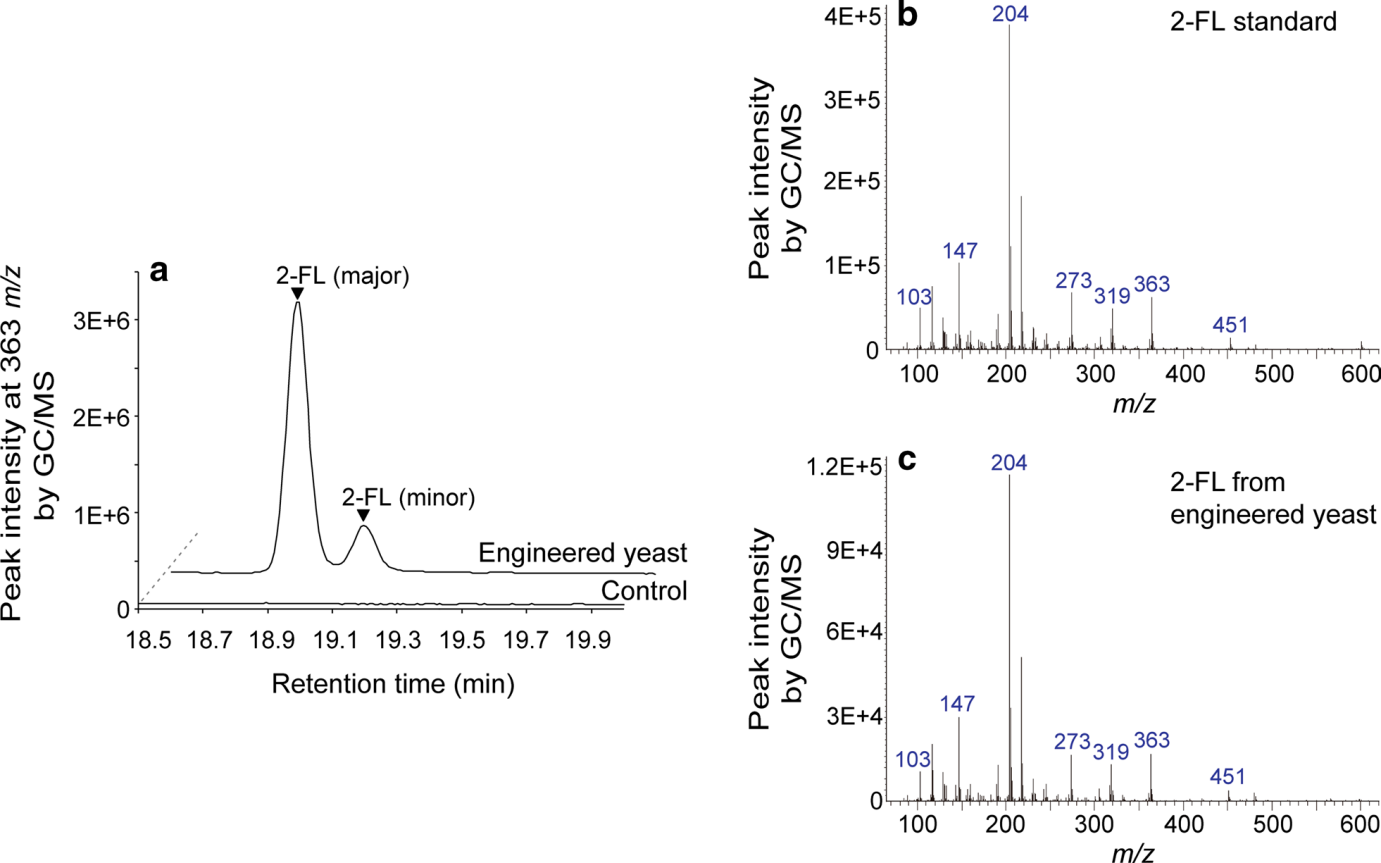
Identification of 2′-fucosyllactose (2-FL) produced by batch fermentation of the engineered yeast *Saccharomyces cerevisiae* D452-2_LFF strain by GC/MS. **a** The overlaid GC/MS chromatogram at 363 *m/z*, showing the unique daughter ions of 2-FL. Mass spectra of **b** 2-FL standard and **c** 2-FL produced by strain D452-2_LFF. *S. cerevisiae* D452-2_LFF, *S. cerevisiae* D452-2 harboring *fkp* coding for l-fucokinase/guanosine 5′-diphosphate-l-fucose phosphorylase, *fucT2* coding for α-1,2-fucosyltransferase, and *LAC12* coding for lactose permease; Control, the D452-2_LFF_Control strain harboring three empty plasmids

To verify the production of 2-FL by the engineered yeast (D452-2_LFF), 2-FL produced in the culture broth was analyzed by GC/MS. The selected ion chromatogram (at 363 *m/z*) of the culture broth of the D452-2_LFF and D452-2_LFF_Control strains indicated that 2-FL was produced only by the engineered *S. cerevisiae* D452-2_LFF (Fig. 4a). By comparing the mass fragmentation patterns of 2-FL standard with its unique fragment ions at 103, 147, 204, 217, 273, 319, and 363 *m/z* (Fig. 4b) and 2-FL synthesized by strain D452-2_LFF (Fig. 4c), the production of 2-FL by the D452-2_LFF strain was verified.

### Fed-batch fermentation for the production of 2-FL by engineered yeast

**Fig. 5:**
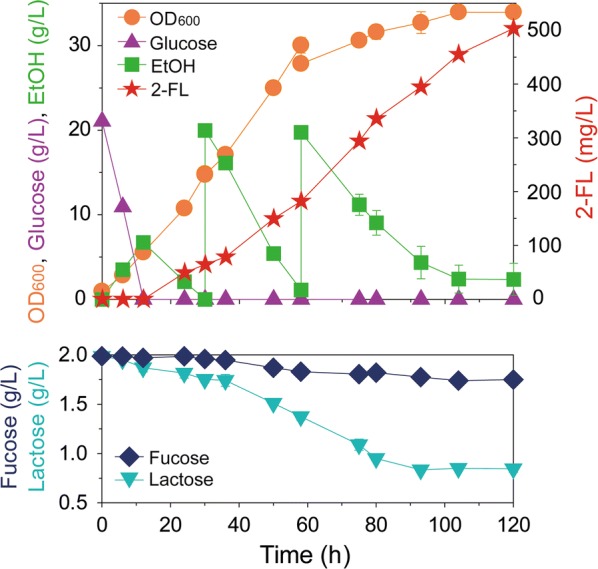
Production of 2′-fucosyllactose (2-FL) by fed-batch fermentation of the engineered *Saccharomyces cerevisiae* D452-2_LFF strain. Ethanol was fed intermittently when depleted. During the fermentation, the cell density (OD_600_) and the concentrations of glucose, ethanol (EtOH), and 2-FL were monitored using HPLC. All points are the means of duplicate experiments. Strain D452-2_LFF indicates *S. cerevisiae* D452-2 harboring *fkp* coding for l-fucokinase/guanosine 5′-diphosphate-l-fucose phosphorylase, *fucT2* coding for α-1,2-fucosyltransferase, and *LAC12* coding for lactose permease

Fed-batch fermentation of strain D452-2_LFF was performed to investigate the feasibility of mass production of 2-FL by the engineered yeast. To increase the titer of 2-FL, the fermentation conditions, such as medium components, temperature, and agitation speed, were maintained as those of batch fermentation, but ethanol was intermittently fed as a carbon source (Fig. 5). Ethanol produced from the initially added glucose was completely consumed at 36 h, and 20 g/L ethanol was added to the flask two times. However, ethanol was not consumed anymore after 120 h. Therefore, the fermentation was stopped, and OD_600_ reached 34.0, and 2-FL concentration reached 503 mg/L at 120 h (Fig. 5). Until 120 h, 270 mg/L of l-fucose and 1.19 g/L of lactose were consumed (Fig. 5). Thus, the final yields of 2-FL were 0.63 mol/mol from l-fucose and 0.3 mol/mol from lactose.

**Fig. 6:**
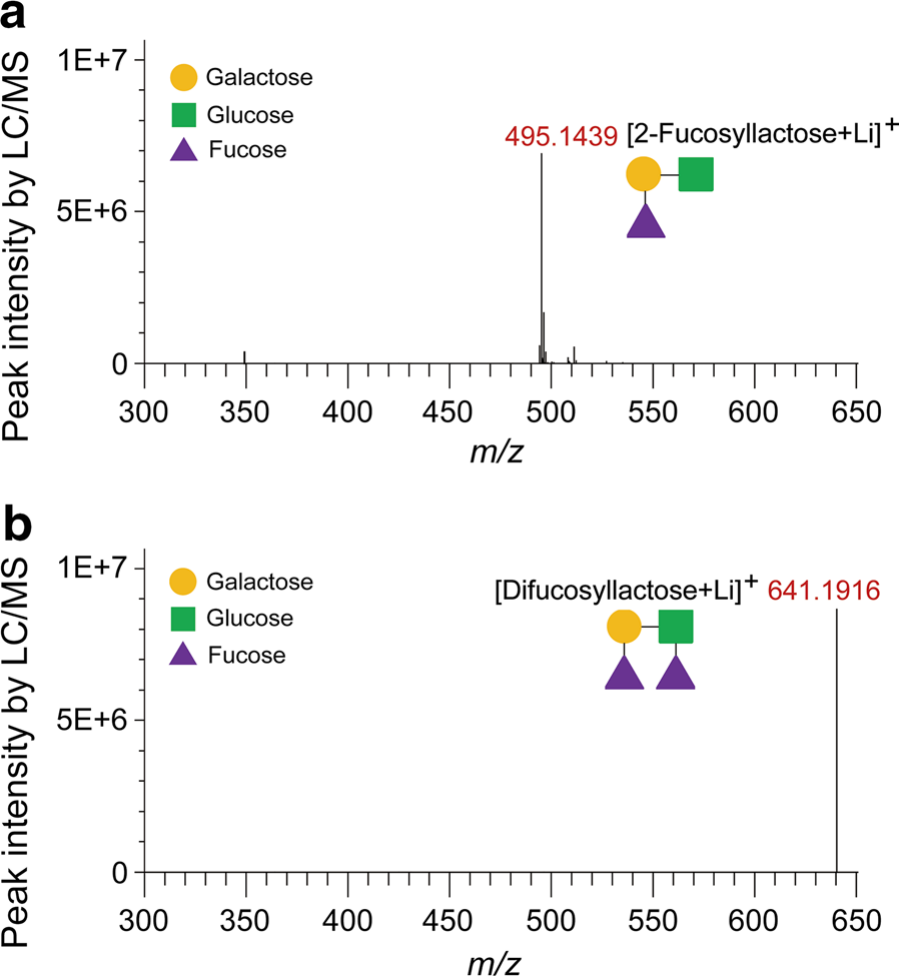
Identification of 2′-fucosyllactose (2-FL) produced by fed-batch fermentation of the engineered yeast, *Saccharomyces cerevisiae* D452-2_LFF. The mass spectra of **a** 2-FL and **b** difucosyllactose produced during fed-batch fermentation were obtained by LC/MS. Strain D452-2_LFF indicates *S. cerevisiae* D452-2 harboring *fkp* coding for l-fucokinase/guanosine 5′-diphosphate-l-fucose phosphorylase, *fucT2* coding for α-1,2-fucosyltransferase, and *LAC12* coding for lactose permease

For the verification of 2-FL produced by fed-batch fermentation, a subsequent analysis of 2-FL was performed by LC/MS. The ion at 495.1439 *m/z* corresponding to 2-FL [(2-FL + Li)^+^] was detected in the culture broth from fed-batch fermentation (Fig. 6a). The production of difucosyllactose was also confirmed by detecting the ion at 641.1916 *m/z* corresponding to difucosyllactose [(difucosyllactose + Li)^+^] (Fig. 6b).

## Discussion

2-FL has been identified as a promising prebiotic in human milk, and economical and safe production of 2-FL on a large scale is highly desirable for various applications in infant formula and foods. *S. cerevisiae* has been widely used as a host for producing pharmaceuticals and nutraceuticals due to its GRAS status and well-developed genetic tools [30, 31]. However, *S. cerevisiae* has not been metabolically engineered to produce 2-FL yet. In this study, for the production of 2-FL, we introduced the 2-FL-producing *salvage* pathway, consisting of three genes from three different microorganisms, into *S. cerevisiae*, and verified 2-FL production in the engineered yeast by GC/MS and LC/MS. To our knowledge, this is the first report of 2-FL production by an engineered yeast.

The first goal of this study was to identify the best FKP to produce GDP-l-fucose in engineered yeast. So far, for the introduction of the *salvage* pathway in *E. coli* and *S. cerevisiae*, the *fkp* gene only from *B. fragilis* 9343 has been tested [14, 16]. Meanwhile, in all *Bacteroides* species, the *salvage* pathway is known to be conserved [10]. Thus, in this study, genes coding for FKP from *Bacteroides* species other than *B. fragilis* 9343 were tested for their efficacies for the production GDP-l-fucose. FKP genes from *B. thetaiotaomicron* and *B. ovatus* were introduced into *S. cerevisiae*, and subsequent production of GDP-l-fucose was confirmed in this study. Nonetheless, the overexpression of *B. fragilis* 9343 *fkp* led to the highest production of GDP-l-fucose in the engineered yeast.

After introducing three genes (*fkp*, *fucT2*, and *LAC12*) which are necessary for the production of 2-FL production into *S. cerevisiae*, 2-FL production by the engineered yeast (D452-2_LFF) was verified in this study. Although this is the first report of 2-FL production by engineered yeast, the titer of 2-FL produced by engineered yeast was much lower than those by engineered *E. coli* in previous reports [9, 11, 14]. In batch fermentation via the *salvage* pathway, only 92 mg/L of 2-FL was produced by the engineered yeast in this study, while 2.08 g/L of 2-FL was produced by *E. coli* [14].

The lower titer of 2-FL by the engineered yeast may be attributed to several reasons. First, it might be due to a lack of suitable transporters for fucose in *S. cerevisiae*. Unlike *E. coli*, *S. cerevisiae* does not possess the fucose metabolic pathway [32]. Therefore, fucose, the substrate for producing GDP-l-fucose, might not be efficiently transported into *S. cerevisiae*, and intracellular fucose concentrations in *S. cerevisiae* might not be high enough to drive efficient phosphorylation of fucose. For instance, after batch fermentation of the D452-2_LFF strain, 285 mg/L of lactose, which was presumed to be transported via Lac12, was consumed after 48 h. However, only 70 mg/L of l-fucose was consumed after 48 h. Therefore, further genetic perturbations to transport l-fucose may be necessary to increase 2-FL production by the engineered yeast.

The second reason for the lower titer of 2-FL produced by the engineered yeast might be the byproduct production during fermentation. We found that not only 2-FL but also difucosyllactose was produced by the engineered yeast. Difucosyllactose is produced by additional fucosylation of 2-FL, which decreases the yield of 2-FL and concentration of intracellular GDP-l-fucose at the same time. Difucosyllactose might have been produced in this study because FucT2, which shows an enzymatic activity towards various substrates [33], was overexpressed for 2-FL production in the engineered yeast. Difucosyllactose production was also observed in engineered *E. coli* [14, 15]. However, when replacing FucT2 with WcfB, a gene coding for α-1,2-fucosyltransferases from *B. fragilis* 9343, difucosyllactose was not produced, and the titer and yield of 2-FL produced by engineered *E. coli* increased [15]. Therefore, it might be possible to increase the titer and yield of 2-FL by overexpressing different α-1,2-fucosyltransferases that do not have a broad substrate specificity, such as WcfB instead of FucT2, in the engineered yeast.

The last reason for the lower titer of 2-FL produced by the engineered yeast might be the problem imposed in exporting 2-FL. As mentioned above, the production of difucosyllactose was observed in this study, implying that 2-FL itself was also used as a fucose acceptor. It can be furthermore speculated that a considerable amount of 2-FL might have remained intracellularly in the engineered yeast without being secreted. To verify this, the intracellular 2-FL concentrations were measured during the fed-batch fermentation in this study. It was found that ~ 25% of 2-FL was found in the fermented media while ~ 75% of 2-FL was found inside cells (Additional file 1: Figure S1). The ratio of extracellular to intracellular 2-FL in the engineered yeast was lower than that ~ 50% obtained by an engineered *E. coli* [13]. Therefore, by improving the export of 2-FL outside cells, the 2-FL production by the engineered yeast could be enhanced.

## Conclusions

As a proof of concept, we have demonstrated for the first time that 2-FL—so far produced by engineered *E. coli*—can also be produced by engineered *S. cerevisiae* via the *salvage* pathway. The 2-FL producing *salvage* pathway was introduced into *S. cerevisiae*, and 2-FL production was verified by GC/MS and LC/MS. Considering numerous benefits of using a GRAS host for mass production, these results have paved a road for more economical and safer production of 2-FL.

## Additional file

**Additional file 1: Figure S1.** Comparison of the volumetric concentrations of extracellular and intracellular 2-FL in a 125-mL flask, produced by fed-batch fermentation of the engineered *S. cerevisiae* D452-2_LFF strain after 30 and 36 h. All data points are the means of experimental data from duplicate fed-batch fermentations. *S. cerevisiae* D452-2_LFF indicates *S. cerevisiae* D452-2 harboring *fkp* encoding L-fucokinase/guanosine 5’-diphosphate-L-fucose phosphorylase, *fucT2* encoding α-1,2-fucosyltransferase, and *LAC12* encoding lactose permease.

## Abbreviations

2-FL: 2′-fucosyllactose
GDP-l-fucose: guanosine 5′-diphosphate-l-fucose
GRAS: generally recognized as safe
GC/MS: gas chromatography/mass spectrometry
HMOs: human milk oligosaccharides
HPLC: high performance liquid chromatography
KHP: potassium hydrogen phthalate
LC/MS: liquid chromatography/mass spectrometry
OD_600_: optical density at 600 nm
RI: refractive index
YSC: yeast synthetic complete
