# Supplementary material for: Production of a human milk oligosaccharide 2′-fucosyllactose by metabolically engineered Saccharomyces cerevisiae
Source: Microb Cell Fact. 2018 Jun 27;17:101. doi: 10.1186/s12934-018-0947-2 (PMC6020385; doi:10.1186/s12934-018-0947-2)
Supplement: Supplementary file 1 — Additional file 1: Figure S1. Comparison of the volumetric concentrations of extracellular and intracellular 2-FL in a 125-mL flask, produced by fed-batch fermentation of the engineered S. cerevisiae D452-2_LFF strain after 30 and 36 h. All data points are the means of experimental data from duplicate fed-batch fermentations. S. cerevisiae D452-2_LFF indicates S. cerevisiae D452-2 harboring fkp encoding L-fucokinase/guanosine 5’-diphosphate-L-fucose phosphorylase, fucT2 encoding α-1,2-fucosyltransferase, and LAC12 encoding lactose permease. [file 12934_2018_947_MOESM1_ESM.doc]

**Additional file 1**


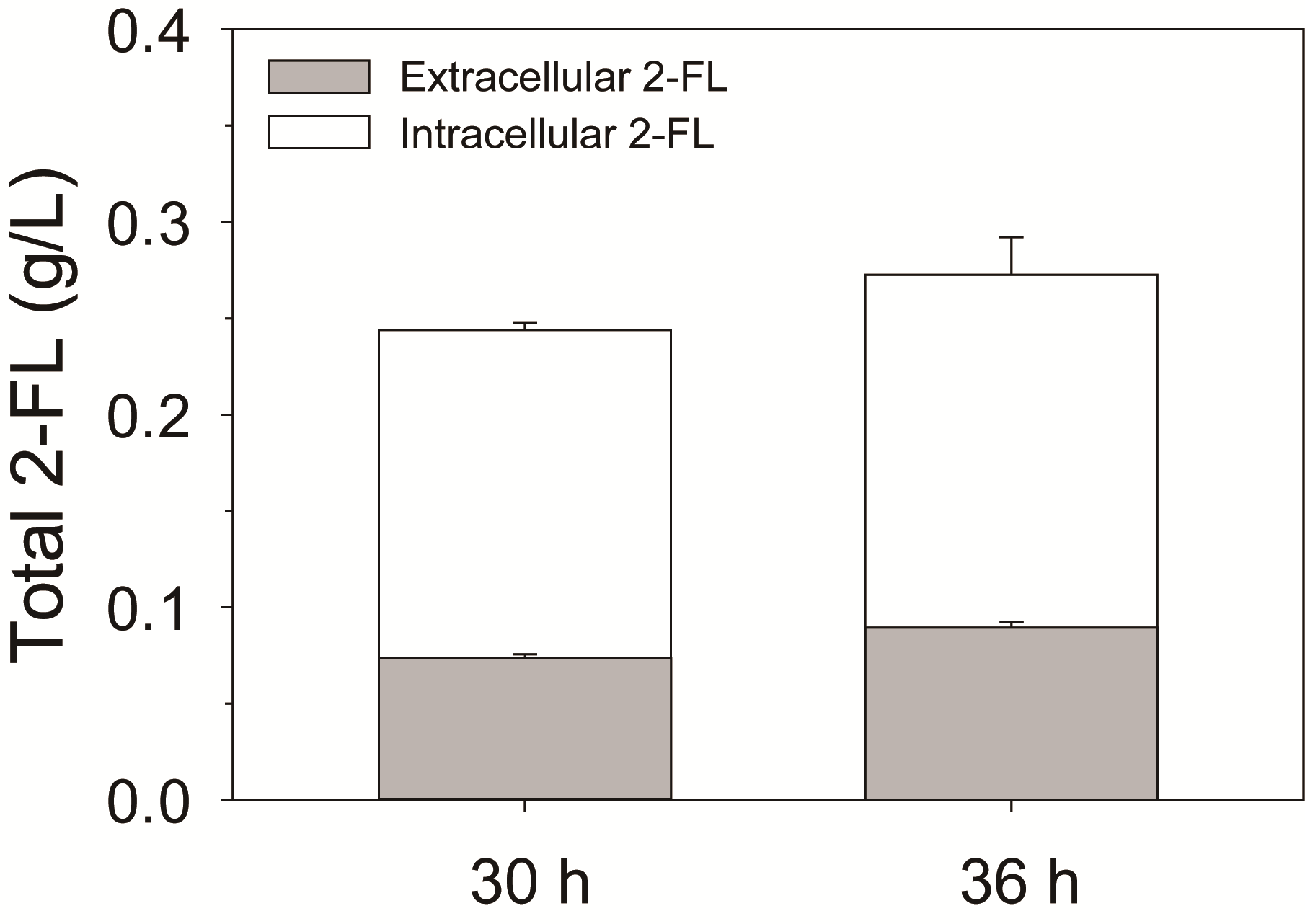


**Figure S1** Comparison of the volumetric concentrations of extracellular and intracellular 2-FL in a 125-mL flask, produced by fed-batch fermentation of the engineered *S. cerevisiae* D452-2_LFF strain after 30 and 36 h. All points are the means of duplicate experiments. *S. cerevisiae* D452-2_LFF is *S. cerevisiae* D452-2 harboring *fkp* encoding L-fucokinase/guanosine 5’-diphosphate-L-fucose phosphorylase, *fucT2* encoding α-1,2-fucosyltransferase, and *LAC12* encoding lactose permease
